# Value of pretreatment 18F-FDG PET/CT in prognosis and the reflection of tumor burden: a study in pediatric patients with newly diagnosed neuroblastoma

**DOI:** 10.7150/ijms.58263

**Published:** 2021-02-24

**Authors:** Shuai Man, Jie Yan, Jie Li, Yanna Cao, Jiajian Hu, Wenchao Ma, Jianjing Liu, Qiang Zhao

**Affiliations:** 1 Department of Pediatric Oncology, Tianjin Medical University Cancer Institute and Hospital, National Clinical Research Center for Cancer, Key Laboratory of Cancer Prevention and Therapy of Tianjin, Tianjin's Clinical Research Center for Cancer, Tianjin, China.; 2 Department of Molecular Imaging and Nuclear Medicine, National Clinical Research Center for Cancer, Tianjin Medical University Cancer Institute and Hospital, Tianjin, Tianjin's Clinical Research Center for Cancer, Tianjin, China.

**Keywords:** 18F-FDG PET/CT, MTV, TLG, neuroblastoma, progression, tumor burden

## Abstract

Fluorine-18 fluorodeoxyglucose (18F-FDG) PET/CT has been commonly used in pediatric patients with newly diagnosed neuroblastoma (NB) for diagnosis. We retrospectively reviewed 40 pediatric patients with newly diagnosed NB who underwent 18F-FDG PET/CT. Clinicopathological factors and metabolic parameters including maximum standardized uptake value (SUVmax), metabolic tumor volume (MTV), and total lesion glycolysis (TLG) on PET/CT were evaluated as predictive factors for progression-free survival (PFS) and overall survival (OS) by univariate and multivariate analysis. Spearman rank correlation analyses were used to estimate the correlations between clinical factors and PET findings. The mean follow-up after 18F-FDG-PET/CT was 32.9 months. During the follow-up period 15 (37.5%) patients experienced progression, and 9 (22.5%) died. MTV (P=0.001) and TLG (p=0.004) remained significant predictive factors for tumor progression, along with lactate dehydrogenase (LDH), neuron-specific enolase (NSE) and bone metastasis. Univariate analysis showed that bone metastasis, LDH (>1064 IU/L), NSE (>364.4 ug/L), MTV (>191 cm3) and TLG (>341.41 g) correlated with PFS, and LDH (>1064 IU/L), NSE (>364.4 ug/L) and MTV (>191 cm^3^) correlated with OS (p<0.05). In multivariate analysis, MTV and bone metastasis were independent prognostic factors for PFS (p=0.001 and 0.023, respectively), and MTV remained the only independent prognostic factor for OS (p= 0.004). We also found that there were correlations between semiquantitative PET/CT parameters and clinical features in NB. Our results suggested that 18F-FDG PET/CT was a useful tool to predictive progression and to reflect tumor burden for patients with NB.

## Introduction

Neuroblastoma (NB) is the most common malignant extracranial solid tumor in paediatric patients that occurs anywhere in the sympathetic nervous system, but mostly occurs in the adrenal medulla, and accounts for 7%-10% of all paediatric tumors 1, 2. Approximately 60% of patients with NB have metastatic disease at presentation. Common metastasis sites include the bone marrow, bone, lymph nodes, and liver 2. More than 50% of patients with NB are diagnosed as high risk with a long-term survival probability < 50% 3. The factors related to prognosis include the age at diagnosis, tumor histology type, tumor biological characteristics, primary tumor site, disease stage, and tumor response to treatment. Based on the risk classification of the Children's Oncology Group (COG), patients were classified into low-risk, intermediate-risk and high-risk groups with different outcomes and different treatment needs 4.

2-deoxy-2-[18F] fluoro-D-glucose (18F-FDG) positron emission tomography/computed tomography (PET/CT) is a non-invasive, whole-body imaging examination that provides complete anatomical information with CT and detects the extent of FDG uptake in the primary tumor and metastases 5, 6. Previous studies have demonstrated the important role of 18F-FDG PET/CT in staging disease, detecting recurrence, and evaluating treatment response for renal cell carcinoma 7, non-small cell lung cancer 8, breast cancer 9, etc. Tumor cells have a higher metabolic activity than normal cells and usually show higher FDG uptake. The most commonly used PET/CT parameter is the maximum standardized uptake values (SUVmax). Some recent studies have shown that metabolic tumor volume (MTV) and total lesion glycolysis (TLG) can provide important information about metabolism and prognosis for various human tumors. There are a few studies on the application of 18F-FDG PET/CT for NB 2, 10, 11. However, most of these studies focused on comparing the diagnostic efficacy of 18F-FDG PET/CT and other imaging methods, such as iodine 123 metaiodobenzylguanidine (I-123 MIBG) 11, 12, or only evaluated the diagnostic value of 18F-FDG PET/CT for NB 13, 14. To the best of our knowledge, there are few studies that have assessed the prognostic significance of the metabolic parameters on PET/CT or have evaluated the correlation between semiquantitative PET/CT parameters and clinical features in patients with NB 15, 16. Therefore, we conducted a retrospective analysis to determine whether 18F-FDG PET/CT can provide prognostic information and reflect tumor burden in NB.

## Materials and methods

### Patients

We retrospectively reviewed the records of all paediatric patients (age < 18 years) with NB who had undergone 18F-FDG PET/CT before treatment from January 2013 to December 2018. The inclusion criteria were as follows: 1) diagnosed by ultrasonography-guided fine-needle aspiration or biopsy and final postoperation pathology and 2) received comprehensive treatment after 18F-FDG PET/CT imaging. The exclusion criteria were as follows: 1) any treatment procedures before 18F-FDG PET/CT investigation and 2) patients without a pathological diagnosis. The data obtained from the clinical medical records included age, sex, risk stratification, MYCN amplification, laboratory test parameters including lactate dehydrogenase (LDH), neuron-specific enolase (NSE), and follow-up information. Next, correlations between initial TLG or SUVmax values and serum markers were investigated in all patients. All patients were followed up for more than 6 months (mean, 32.9 months; range, 6-77 months). Patients were classified to progression group and the non-progression group. Local tumor progression, tumor recurrence, metastasis and death due to the disease were considered as progression. Recurrence referred to the complete disappearance of the tumor 1 month later with local or metastatic tumor lesions that appeared again. Metastatic lesions found on any image throughout the study period were recorded. BMI was verified by bone marrow biopsy (BMB). Metastatic lesions found on any image, such as CT, magnetic resonance imaging (MRI) and bone scan, throughout the study period were recorded.

This study protocol was approved by the institutional review board of Tianjin Medical University Cancer Institute and Hospital. All methods were carried out in accordance with the relevant guidelines and regulations.

### 18F-FDG PET/CT

The NB patients fasted for at least 6 hours prior to the examination and had a blood glucose lower than 10 mmol/L. The intravenous injection of FDG ranged from 4.44-5.55 MBq/kg, depending on the body surface area. Twenty-three patients were given oral or i.v. sedation for PET scans. PET/CT examinations were performed 60 minutes after injection of the tracer using a GE PET/CT system (Discovery 690). The examinations included a head-to-toe CT scan (80 kV; 50-100 mAs.) and a 3-dimensional (3D) PET scan (2 minutes per bed, 6-7 beds). The rotation time was 0.6. The slice thickness was 3.75 mm. The increment was 3.27. The pitch was 0.984. The images were displayed on the Xeleris workstation.

### Image analysis

Two experienced nuclear medicine physicians reviewed the 18F-FDG PET/CT images independently. Depending on the axis and the coronal and sagittal projections, the physicians placed the volume of interest (VOI) on the primary tumor. The metabolic parameters, such as SUVmax, SUVmean and MTV, were measured on PET/CT images using volume viewer software, and TLG was then calculated as [TLG = SUVmean × MTV]. In our study, we selected an SUVmax of 42% as the threshold for generating the VOI.

### Statistical analysis

Statistical analyses were performed using SPSS software (version 25.0 for Windows; SPSS INC.). Continuous data are described as the mean ± standard deviation (mean ± SD) or median and range, depending on whether they followed a normal distribution, and categorical variables are described as numbers.

- PET/CT parameters and clinicopathological factors were compared between the progression group and the non-progression group using independent t-tests, Mann-Whitney U tests, χ2 tests, and Fisher exact tests.
- Spearman rank correlation analyses were used for to analyse the relationship between serum biomarker levels (LDH, NSE) and semiquantitative PET/CT parameters (SUVmax, MTV, TLG). The relationships among MYCN amplification, risk stratification, lymph node metastasis, and PET/CT parameters were analysed with t-tests or Mann-Whitney U tests.
- Progression-free survival (PFS) and overall survival (OS) were calculated to evaluate the risk of progression and death. PFS was defined as the time from the day of diagnosis to the first documented evidence of disease progression or recurrence, based on any radiographic examination. OS was defined as the time from the day of diagnosis to the date of death or last follow-up. Survival curves were estimated using the Kaplan-Meier method to calculate cumulative PFS rates and OS rates and compared with the log-rank test.
- Receiver operating characteristic (ROC) curve analysis was used to find the best cut-off value to classify LDH, NSE, SUVmax, MTV and TLG using Cox proportional hazards regression. These parameters and bone metastasis were assessed separately for the univariate analysis and multivariate analysis for their ability to predict OS.
- The uptake in the bone marrow on 18F-FDG PET/CT images, as determined by the SUVmax and the different patterns of lesions between the positive bone marrow biopsy group and the negative group, were estimated using Mann-Whitney U tests and χ^2^ tests, respectively.

All tests were two-sided, and a probability of less than 0.05 was considered statistically significant.

## Results

We retrospectively reviewed the records of sixty-eight paediatric patients (age < 18 years) and finally 40 patients (31 at high-risk) were the subjects of this study. This study consisted of 21 females and 19 males with an average age of 37.78 months. All patients received standardized treatment according to their risk group. During the clinical follow-up period, 15 (37.5%) patients experienced progression, among whom 9 (22.5%) died. The characteristics of the patients are shown in **Table 1.**

### Predictive efficacy of NB between progression and non-progression

In our study, there were significant differences in bone metastasis, NSE, LDH, MTV and TLG between patients with progression and those without; age, sex, MYCN amplification, GD2 expression, tumor location, lymph node metastasis, other distant metastasis, bone marrow involvement, risk stratification and SUVmax showed no significant differences between the two groups (**Table 1**).

### Survival analysis

Age, LDH, NSE, bone metastasis, BMI, SUVmax, MTV and TLG were evaluated as variables in the survival analysis of patients with NB.

#### Univariate and multivariate analysis for PFS

**Figure 1:**
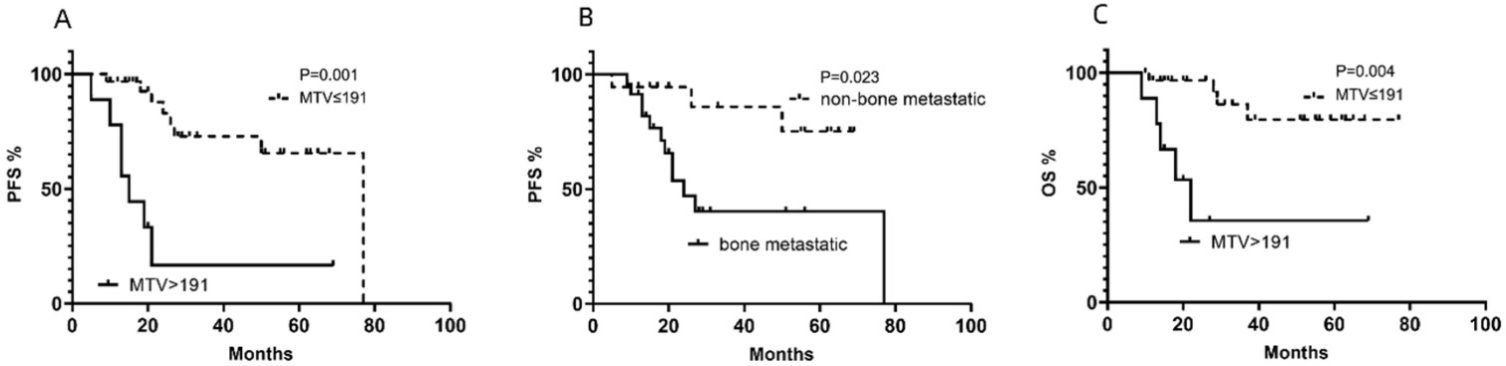
Cumulative progression-free survival (PFS) curves according to (**A**) metabolic tumor volume (MTV) and (**B**) bone metastasis and (**C**)cumulative overall survival (OS) curve according to MTV of NB lesions in enrolled patients.

In the univariate analysis of PFS, LDH, NSE, TLG, MTV and bone metastasis were determined to be statistically significant factors for PFS, although the effects of some of these factors were estimated to be relatively small (**Table 2**). In the multivariate analysis of PFS, MTV (**Fig. 1A**) and bone metastasis (**Fig. 1B**) were significant risk factors for PFS (**Table 2**).

#### Univariate and multivariate analyses for OS

**Figure 2:**
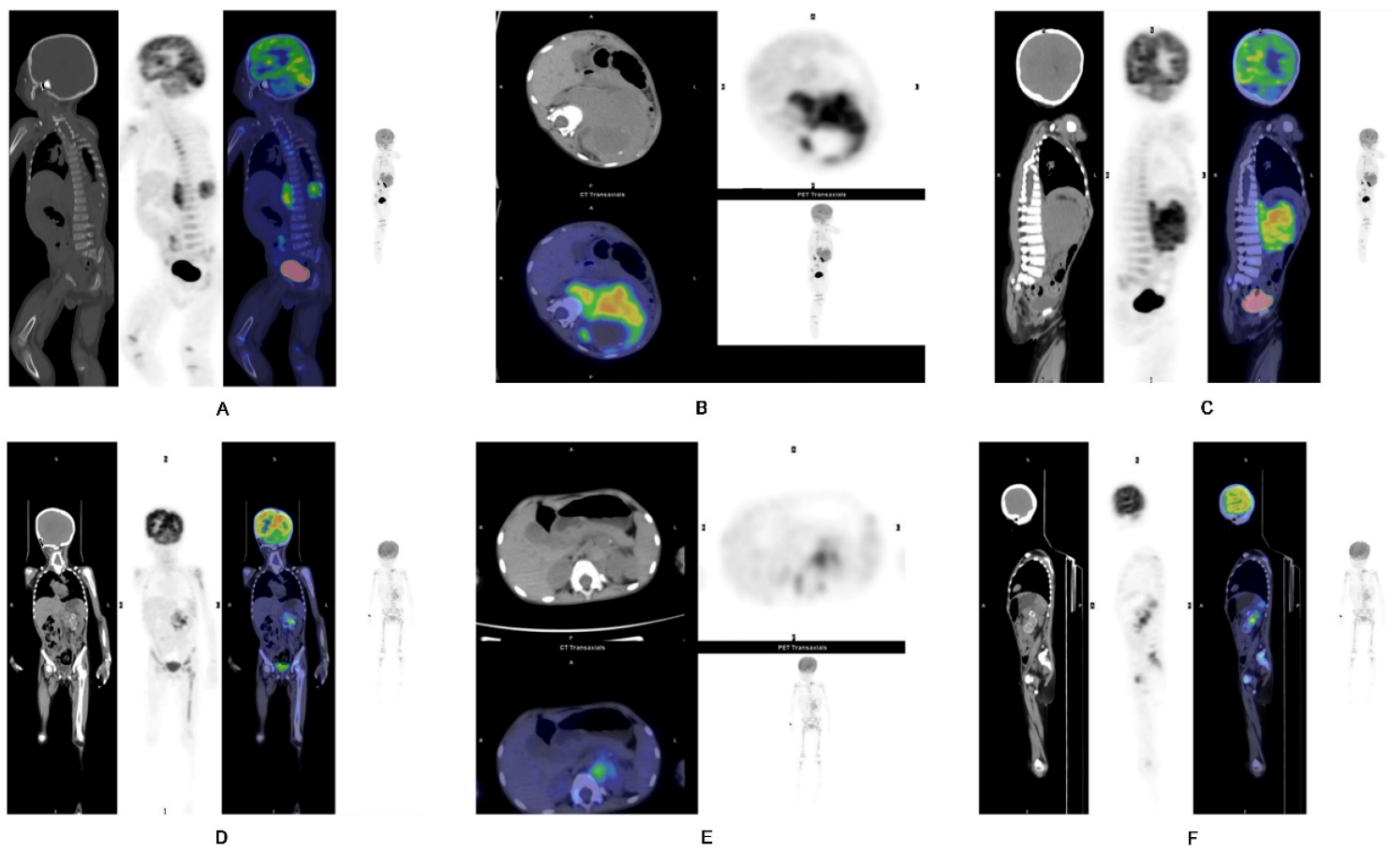
** A-C)** Representative images of a 1-year-old boy with stage IV NB. PET/CT revealed a primary tumor at the left suprarenal region, seen in the (A) coronal, (B) axial and (**C**) sagittal views. Intense metabolic tumor volume (MTV) (236) of the primary tumor was noted on the axial image. He progressed and eventually died. **D-F)** Representative images of a 4-year-old girl with stage IV NB. PET/CT revealed the primary tumor at left suprarenal region, seen in the (**D**) coronal, (**E**) axial, and (**F**) sagittal views. Small metabolic tumor volume (MTV) (46.9) of the primary tumor was noted on the axial image. She has not progressed yet.

From the ROC analyses, the optimal cutoff values for LDH, NSE, SUVmax, MTV and TLG were 1064 IU/L, 364.4 IU/L, 12.01, 191 cm^3^, and 341.41 g, with AUCs of 0.7238, 0.7410, 0.4854, 0.7148 and 0.6661, respectively. LDH, NSE and MTV showed significant differences in the univariate analysis of OS. In the multivariate analysis of OS, MTV (**Fig. 1C**) was the only significant risk factor for OS (**Table 3**). MTV was the only independent prognostic factor for both PFS and OS (**Fig. 2**) and other clinical characteristics or metabolic 18F-FDG PET/CT features were significantly associated with the survival outcomes for patients with NB (**Tables 2 & 3**).

### Correlation between semiquantitative PET/CT parameters and clinical features

#### Lymph node metastasis analysis

Compared with patients without lymph node metastasis, patients with lymph node metastasis had higher TLG values (p=0.048<0.05).

#### BMI analysis

**Figure 3:**
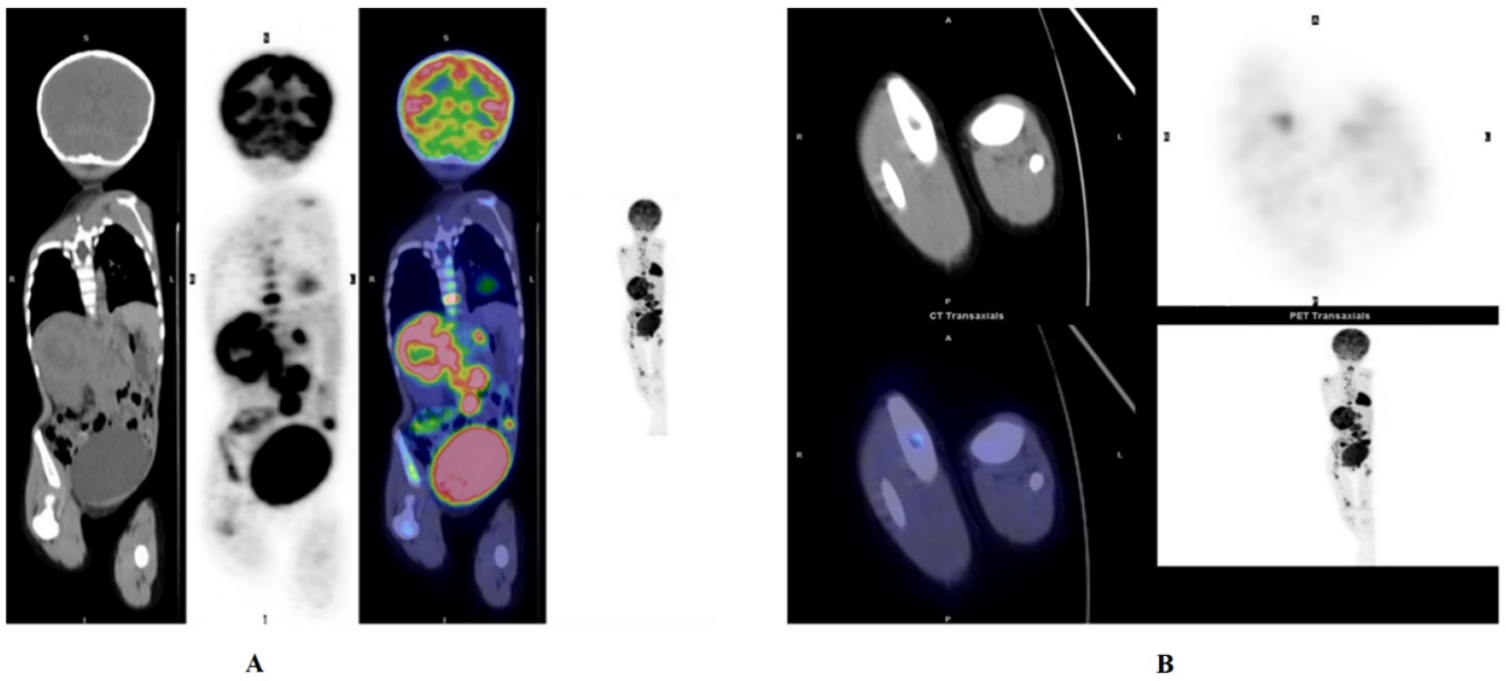
NB. Whole body PET/CT shows NB in the left adrenal gland. Bone marrow infiltration in the left and right femur head as well as the proximal tibia. (**A**) Activity in the right pelvis was observed. (**B**)Bone marrow infiltration in the left and right femurs. This patient had a large SUVmax-ratio value, 8.781, and eventually the patient progressed, and eventually died, with very short progression-free survival (PFS) and overall survival (OS).

Before systemic therapy, unilateral or bilateral blind BMB of the posterior iliac crest were routinely performed in 40 patients, and the BMIs were verified in 29 patients. When analysing the PET/CT results of 40 patients, we recorded the bone marrow SUVmax of 40 patients, and their liver SUVmax and lesion-to-liver ratio (SUVmax-ratio) were calculated. We found that patients with positive BMBs had higher bone marrow SUVmax measurements than those with negative BMBs (t=3.567, p≤0.001). From the ROC analyses, the optimal cutoff value for the SUVmax-ratio was 1.39, with an AUC of 0.703. The larger the SUVmax-ratio was, the worse the outcome (z=2.123, p=0.034) (**Fig. 3**). In other words, the SUVmax-ratio can be further used to assess prognosis.

#### COG risk classification

Forty paediatric patients with NB were divided into a high-risk group and a non-high-risk group. Patients in the high-risk group had higher TLG than those in the non-risk group (t=3.224, p=0.003).

#### Serum biomarker levels

**Figure 4:**
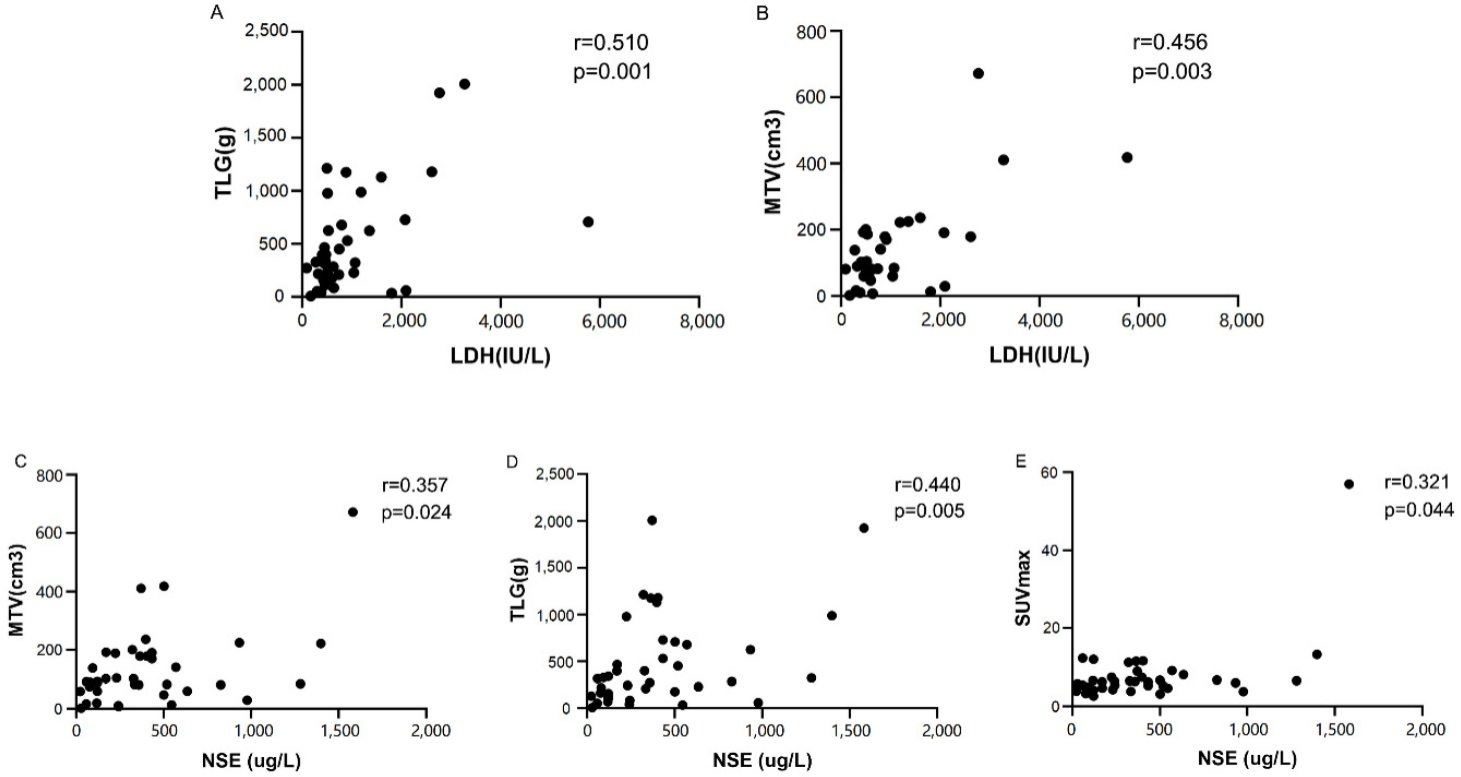
Spearman correlation analyses indicating a strong correlation between lactate dehydrogenase (LDH) and (**A**) total lesion glycolysis (TLG) or (**B**) metabolic tumor volume (MTV). Spearman correlation analyses indicating a strong correlation between the neuron‑specific enolase (NSE) level and (**C**) metabolic tumor volume (MTV), (**D**) total lesion glycolysis (TLG) or (**E**) SUVmax

Spearman analysis showed that the semiquantitative PET/CT parameters were positively correlated with the serum biomarkers levels (**Fig. 4**), except for SUVmax and LDH (p=0.153) (**Table 4**). TLG, SUVmax, and MTV were compared separately based on the median of LDH (594 IU/L) and NSE (333.6 ug/L). The Mann-Whitney U tests showed that compared with patients with low NSE levels, patients with high NSE levels had significantly higher TLG (p=0.008). There was a tendency for correlations between NSE and MTV and SUVmax (p=0.056 and p=0.072, respectively). There was also a tendency for a correlation between the LDH level and TLG (p=0.068). However, no such correlation was found between the LDH level and MTV (p=0.238).

## Discussion

Evaluations to determine the disease stage in children with NB commonly includes imaging of the primary tumor site with CT or MRI to determine the primary tumor size and regional invasion and spread 1. Although these methods can provide accurate anatomical information of the tumor, due to the metabolism of the tumor tissue and the surrounding tissue of the tumor, it is difficult and insufficient to accurately assess the true tumor volume with these methods. The current mainstream international functional imaging methods for NB include 18F-FDG PET/CT, I-123 MIBG and fluorine-18 fluorodeoxyphenylalanine (18F-DOPA) PET/CT. Unfortunately, I-123 MIBG and 18F-DOPA PET/CT are unavailable or less applicable in some countries. Because of the increased metabolism and accelerated proliferation, the tumor tissue consumes abundant glucose, which is manifested as increased FDG uptake 17. Thus, the extent of FDG uptake can provide information about tumor disease activity. Previous studies have mostly focused on the diagnostic efficacy and significance of 18F-FDG PET/CT for staging 11, 12, 18-21; however, to date, the prognostic value of this method is still not completely clear. Chao Li et al. 16 concluded that during subsequent treatment, patients with high focal bone marrow uptake and MTV and TLG on 18F-FDG PET/CT may have clearly inferior outcomes. In the present study, we enrolled 40 pediatric patients with newly diagnosed NB to evaluate the prognostic value 18F-FDG PET/CT.

Our results suggest that MTV, TLG, serum levels of LDH and NSE, and bone metastasis are important factors for survival. SUVmax, MTV and TLG have been recognized as prognostic factors in many malignancies 7, 21, 22. The long-term follow-up analysis of the 40 patients in our study showed that the most discriminative MTV cut-off value had prognostic merit and MTV was a significant independent prognostic factor. MTV had a stronger association with both PFS and OS than other clinicopathological factors and metabolic parameters, which is similar to results of other studies 22, 23. The results suggest that MTV may be an important factor during of planning treatment and the follow-up of NB patients.

In approximately 70% of patients, metastasis is present at the time of diagnosis and most commonly involves cortical bone and bone marrow 24. BMI correlates with clinical stage and prognosis and is a frequent site of disease recurrence 25. The detection of BMI also plays important role in predicting progression. In the present study, bone marrow SUVmax and the SUVmax-ratio were two meaningful parameters for NB patients with BMI. PET/CT has high sensitivity for assessing bone marrow infiltration in pediatric malignancies 13, 26. In particular, PET has improved sensitivity in detecting BMI in patients with lymphoma and may allow patients with positive BMI confirmed by PET/CT examinations to avoid undergoing invasive BMB 27, 28. However, there is no clear conclusion on the significance of PET/CT for assessing the metabolism of tumor cells in the bone marrow of patients with NB. An important advantage of PET/CT over BMB is that PET/CT can assess all bone marrow sites at once and find unintended bone marrow infiltration in areas where biopsies are not usually performed 13. The bone marrow manifests higher metabolic activity and FDG uptake when tumor cells have transferred to the bone marrow, which can guide clinical diagnosis, staging and treatment. Because of its consistent results with BMB and its prognostic value in predicting progression, PET/CT has potential advantages as a non-invasive systemic examination. Our study demonstrated that the uptake intensity in the bone marrow may have significant prognostic implications and that 18F-FDG PET/CT has a substantial role in initially determining the BMI in NB patients. Additionally, the routine use of BMB of the posterior iliac crest may be reconsidered when 18F-FDG PET/CT is available. Considering the small number of cases, to some extent, this exploration was limited, and the bone marrow itself has physiological uptake and inflammation and several drugs can affect FDG PET/CT examinations. Therefore, whether 18F-FDG PET/CT can provide more information on BMI for patients with NB, or even replace BMB still requires a larger number of clinical cases and more in-depth studies.

Serum LDH and NSE tests are simple, and these levels can be used as useful prognostic markers for NB 29-32. However, serum LDH lacks the sensitivity and specificity to monitor disease activity. Serum NSE is more specific than LDH, but its sensitivity is still low. Regarding serum biomarkers, we demonstrated in this study that the serum LDH and NSE levels strongly correlated with TLG and MTV in 39 patients (the serum biomarker data of one of the forty patients were not available). This finding can be explained by the fact that LDH is related to the glucose metabolism of tumor cells. Previous research has shown that LDH is associated with the aerobic or anaerobic pathways of glycolysis 33. In addition, the more important reason is that tumor markers such as serum biomarkers and metabolic parameters determined by 18F-FDG PET/CT can both represent tumor burden. This result also showed that, compared with SUVmax, MTV and TLG of the primary tumor can reflect the metabolic burden of tumor lesions more accurately. Previous studies showed that MTV and TLG could represent a combination of tumor volume and metabolism, including both morphological and metabolic features, and may be considered an expression of tumor aggressiveness in addition to tumor size 34. Thus, 18F-FDG PET/CT, as a very suitable functional imaging method, can represent the tumor burden 35.

Tumor burden is often related to the tumor's response to treatment and has a significant impact on prognosis. Therefore, finding a method to accurately assess the tumor burden of pediatric patients with NB is important and urgent. The combined analyses of clinical characteristics, FDG uptake in the bone marrow and differences in survival outcomes between progressive and non-progressive patients showed that the metabolic parameters determined by 18F-FDG PET/CT can be a good representation of the tumor burden for pediatric patients with NB. Meanwhile the assessment of disease burden is also vital to the identification of progression 36.

Our research has several limitations. The statistical power was largely limited by the small number of subjects and the significance of the volumetric parameters might be more prominent with a larger sample size, considering the bulky and heterogeneous nature of NB. The tumor burden assessment based on the segmentation was performed only in primary tumor because the application of the technology to evaluate total tumor burden in children's solid tumors is difficult. On the other hand, NB tends to metastasize to the skull/skull base and the area is difficult to assess with FDG-PET due to the high FDG uptake in the brain. In some cases, a misestimation of the metabolic signal may interfere with the evaluation.

## Conclusions

The metabolic parameters determined by pretreatment 18F-FDG PET/CT are a significant reflection of tumor burden and have predictive value for patients with NB. TLG and MTV, in particular, are very important. MTV and TLG are related to several clinical characteristics of NB, and they can express the tumor burden of NB; additionally, larger values of MTV and TLG may indicate disease progression. MTV is the only independent prognostic factor for both PFS and OS among SUVmax, TLG, and other prognostic factors. Patients with a higher MTV may have worse outcomes, including a shorter PFS and OS. Bone marrow SUVmax is also of great significance for the prognosis of NB. Pretherapeutic metabolic parameters determined by18F-FDG PET/CT may play important roles in the diagnosis and prognosis of paediatric patients with NB, and large-scale prospective studies are also needed to validate the results by longer follow-up.

## Figures and Tables

**Table 1:** Comparison of clinical characteristics and pretreatment PET/CT findings between progression and non-progression patients

Characteristic	Total (n= 40)	Non-progression (n=25)	Progression (n=15)	*p*
**Sex**	40			0.462
Female		12	9	
Male		13	6	
**Age (month)**	40		15	0.426
Mean±SD (range)	37.78±22.958 (6-84)	35.400±21.670 (6-80)	40.733±25.226(8-84)	
**Tumor location**	40			0.151
Retroperitoneal		21	10	
Mediastinal		4	3	
Other location		0	2	
**Bone metastasis**	40			0.014
Yes		10	12	
No		15	3	
**Lymph node metastasis**	40			0.123
Yes		14	12	
No		11	3	
**Other distant metastases^a^**	40			0.182
Yes		8	8	
No		17	7	
**BMI**	40			0.158
Yes		16	13	
No		9	2	
**Risk stratification**	40			0.063
No-high risk		8	1	
high-risk		17	14	
**MYCN amplification**	36^a^			0.107
Yes		3	5	
No		20	8	
**NSE (ug/L)**	39^b^	25	14	0.006
Median	333.6	240.2	467.25	
Range	22.81-1582	22.81-1282	120.8-1582	
**LDH (IU/L)**	39^b^	24	15	0.011
Median	594	487.5	911	
Range	88.2-5767	88.2-2610	430-5767	
**NSE (ug/L)**	39^b^	25	14	0.006
Median	333.6	240.2	467.25	
Range	22.81-1582	22.81-1282	120.8-1582	
**SUVmax**	40	25	15	0.804
Median	6.2	6.23	5.96	
Range	2.63-57.03	2.63-12.32	3.14-57.03	
**TLG (g)**	40	25	15	0.004
Median	325.43	227.25	626.97	
Range	6.39-2009.28	6.39-1179.97	58.62-2009.28	
**MTV (cm^3^)**	40	25	15	0.001
Median	92.11	82.44	189	
Range	1.74-672	1.74-192	29.14-672	

^a^ distant metastasis except for bone marrow or bone; ^b^ Some data is not available. NSE, neuron‑specific enolase; LDH, lactate dehydrogenase; BMI, bone marrow involvement; SUVmax, maximum standardized uptake values; TLG, total lesion glycolysis; MTV, metabolic tumor volume.

**Table 2:** Univariate and multivariate analysis of prognostic factors for PFS

Variable	Univariate analysis		Multivariate analysis	
	HR (95% CI for HR)	*p*	HR (95% CI for HR)	*p*
Age (month)	0.998 (0.976-1.019)	0.827		
LDH (IU/L)	1.001 (1.000-1.001)	0.001		
NSE (ug/L)	1.001 (1.000-1.002)	0.02		
SUVmax	1.024 (0.983-1.065)	0.254		
TLG (g)	1.001 (1.000-1.002)	0.005		
MTV (cm^3^)	1.004 (1.002-1.007)	0.002	6.820(2.093-22.223)	0.001
Bone metastasis	3.981 (1.094-14.49)	0.036	4.841(2.106-18.759)	0.023
BMI	2.462 (0.547-11.081)	0.241		
Risk classification	4.323 (0.565-33.073)	0.158		

LDH, lactate dehydrogenase; NSE, neuron specific enolase; SUVmax, maximum standardized uptake values TLG, total lesion glycolysis; MTV, metabolic tumor volume; BMI, bone marrow involvement.

**Table 3:** Univariate and multivariate analysis of prognostic factors for OS

Variable	n	Percent	Univariate analysis		Multivariate Analysis	
			HR (95% CI for HR)	P	HR (95% CI for HR)	P
**LDH (IU/L)**	39		5.858(1.514-22.662)	0.01		
≤1064	29	74.4%				
>1064	10	25.6%				
**NSE (ug/L)**	39		5.143(1.064-24.867)	0.042		
≤364.4	22	56.41%				
>364.4	17	43.6%				
**TLG(g)**	40		2.560(0.639-10.259)	0.184		
≤341.41	21	52.5%				
>341.41	19	47.5%				
**MTV (cm^3^)**	40		8.221(2.048-32.996)	0.003	7.772(1.939-31.159)	0.004
≤191	31	77.5%				
>191	9	22.5%				
**Bone metastasis**			3.280(0.678-15.877)	0.140		
Yes	22	55%				
No	18	45%				

LDH, lactate dehydrogenase; NSE, neuron specific enolase; TLG, total lesion glycolysis; MTV, metabolic tumor volume.

**Table 4:** Spearman analysis between metabolic parameters of 18F-FDG PET/CT and serum biomarker levels

Parameters	LDH (IU/L)		NSE (ug/L)	
	r	*p*	r	*p*
SUVmax	0.23	0.153	0.321	0.044
TLG (g)	0.51	0.001	0.440	0.005
MTV (cm^3^)	0.456	0.003	0.357	0.024

SUVmax, maximum standardized uptake values; TLG, total lesion glycolysis; MTV, metabolic tumor volume; LDH, lactate dehydrogenase; NSE, neuron specific enolase.

## Abbreviations

18F-FDG: 2-deoxy-2-[18F] fluoro-D-glucose
PET/CT: positron emission tomography/computed tomography
NB: neuroblastoma
SUVmax: maximum standardized uptake value
MTV: metabolic tumor volume
TLG: total lesion glycolysis
PFS: progression-free survival
OS: overall survival
LDH: lactate dehydrogenase
NSE: neuron-specific enolase
COG: Children's Oncology Group
I-123 MIBG: iodine 123 metaiodobenzylguanidine
CR: complete remission
VGPR: very good partial response
BMI: bone marrow involvement
BMB: bone marrow biopsy
VOI: volume of interest
HL: Hodgkin's lymphoma
18F-DOPA: fluorine-18 fluorodeoxyphenylalanine
